# Sophisticated lessons from simple organisms: appreciating the value of curiosity-driven research

**DOI:** 10.1242/dmm.031203

**Published:** 2017-12-01

**Authors:** Robert J. Duronio, Patrick H. O'Farrell, Greenfield Sluder, Tin Tin Su

**Affiliations:** 1 Departments of Biology and Genetics, Integrative Program for Biological and Genome Sciences, and Lineberger Comprehensive Cancer Center, UNC Chapel Hill, NC 27599-3280, USA; 2 Department of Biochemistry and Biophysics, University of California, San Francisco, CA 94158-2517, USA; 3 Department of Radiology, University of Massachusetts Medical School, Worcester, MA 01655, USA; 4 Department of Molecular Cellular and Developmental Biology, University of Colorado, Boulder, CO 80309-0347, USA

**Keywords:** *C. elegans*, *Drosophila*, Invertebrates, Sea urchin, Yeast

## Abstract

For hundreds of years, biologists have studied accessible organisms such as garden peas, sea urchins collected at low tide, newt eggs, and flies circling rotten fruit. These organisms help us to understand the world around us, attracting and inspiring each new generation of biologists with the promise of mystery and discovery. Time and time again, what we learn from such simple organisms has emphasized our common biological origins by proving to be applicable to more complex organisms, including humans. Yet, biologists are increasingly being tasked with developing applications from the known, rather than being allowed to follow a path to discovery of the as yet unknown. Here, we provide examples of important lessons learned from research using selected non-vertebrate organisms. We argue that, for the purpose of understanding human disease, simple organisms cannot and should not be replaced solely by human cell-based culture systems. Rather, these organisms serve as powerful discovery tools for new knowledge that could subsequently be tested for conservation in human cell-based culture systems. In this way, curiosity-driven biological research in simple organisms has and will continue to pay huge dividends in both the short and long run for improving the human condition.

## Abstract (precis)

**Summary:** Curiosity-driven research using simple organisms has and will continue to lead to fundamental discoveries about biology that are directly applicable to improving the human condition.

## Introduction

The emphases and directions of medical practice are influenced not only by scientific evidence but also by factors such as financial interests and societal trends. For example, interventions such as cupping, the application of suction cups to the skin, have gained popularity not due to scientific evidence (Lee et al., 2011), but based on popular figures promoting the practice. In contrast, impactful progress in medicine invariably follows major advances in biological understanding due to scientific discovery. Examples extend from Pasteur's recognition of the infectious basis of many diseases to the recent discovery of CRISPR/Cas9-based genome editing (see Box 1 for a glossary of terms) (McNutt, 2015; Porter, 1961). Such advances in our understanding of biology have driven revolutionary advances in medicine, in addition to the incremental and unsteady progress that medicine typically makes. Despite this history of success, science is increasingly being tasked to focus on application rather than discovery (Fang and Casadevall, 2010; Hand et al., 2013; Minogue and Wolinsky, 2010). It is therefore with a sense of urgency that we write this article to defend the winning strategy of ‘discovery first’.

**Box 1. Glossary**

**Centrosomes:** multi-protein structures that determine spindle polarity in mitosis through their function as primary microtubule-organizing centers of the cell.

**Chromatin:** DNA complexed with associated proteins, particularly histones.

**Cleavage furrow:** the constriction that cleaves the cell between the separated sister chromosomes at the end of mitosis.

**Conditional mutants:** mutants that show the phenotype of interest only under specific conditions.

**CRISPR/Cas9:** clustered regularly interspaced short palindromic repeat (CRISPR)/CRISPR-associated protein 9. This term is used to refer to a genome editing tool.

**Differential interference contrast (DIC):** a type of microscopy that uses polarized light to distinguish subcellular features in transparent objects.

**Epigenetic regulation:** alteration in gene transcription due to changes in chemical modification of DNA or associated proteins.

**Epistasis:** a genetic analysis method in which the phenotype of a mutant defective in two genes of interest is compared to the phenotype of single mutants in each gene. The results can be used to infer the order in which the products of the two genes act in a biological pathway.

**Genetic screen:** an experimental technique in which individuals are selected from a mutagenized population based on a specific phenotype.

**Mechanical fragmentation:** method used to cut sea urchin zygotes into viable fragments containing different nuclear makeups to, for example, dissect the contribution of nucleus vs cytoplasm.

**Mendelian ratio:** the ratio of genotypes or phenotypes in the progeny when a trait is inherited according to the law of Mendel.

**Mendelism:** inheritance of traits according to the laws of Mendel.

**Microinjection:** injection of foreign substances into cells. Microinjection of sea urchin zygotes can be used, for example, to knock down gene expression with antisense morpholino oligonucleotides.

**Model organism:** experimentally tractable organism used to understand fundamental biological mechanisms that also apply to other organisms of interest.

**Organoid:** an *in vitro* 3D cellular cluster derived exclusively from primary tissue, embryonic stem cells or induced pluripotent stem cells (iPSCs) that is capable of self-renewal and self-organization, and that exhibits similar organ functionality as the tissue of origin. Definition accredited to Fatehullah et al., 2016.

**Polyspermy:** fertilization with more than one sperm per egg.

**Position effect variegation (PEV):** a phenotype whereby cells with the same genotype sometimes exhibit different phenotypes because of transcriptional inactivation of a gene that is abnormally juxtaposed to heterochromatin.

Our understanding of human diseases and the ability to treat them hinges on a foundation of knowledge about basic biology. This knowledge has been gained largely by research using organisms that are experimentally tractable in ways that humans or human cells can never be. Those who do not know the history of yeast, flies and other non-vertebrate organisms and their contributions to biomedicine may consider such studies as unworthy of participation or of funding. Given the growing excitement about human stem-cell-based cell culture systems, particularly organoids (Box 1), some may argue that organisms that do not recapitulate all of the complexity of humans will cease to be useful in the near future. This line of thought is not only incorrect but could hamper scientific progress.

In this article, we highlight the continuing value of curiosity-driven research, with a focus on widely used non-vertebrate experimental organisms. These are often referred to as ‘model organisms’ (Box 1) to highlight their utility in discovering and understanding fundamental biological principles that also apply to other organisms, particularly humans, and to mechanisms of disease. Although we use this term for the remainder of the article, we wish to emphasize that research using such organisms need not be motivated solely for the purpose of modeling human biology in order to significantly enhance our understanding of human disease.

We will illustrate the utility of each model organism with seminal historical examples, rather than providing a comprehensive overview. We hope to motivate the reader to look into additional contributions. Indeed, it is hard to think of an aspect of biology that has not benefited from studies in model organisms, be it behavior (e.g. Kravitz and Fernandez, 2015), aging (e.g. Kenyon, 2011) or memory (e.g. Dubnau and Tully, 1998). Highlighting past achievements will not necessarily encourage future work. Therefore, we also discuss potential future breakthroughs that might come from curiosity-driven research in model organisms. This is not to say that we disagree with the well-supported contention that no one can predict at the time of discovery how applicable findings from basic research will turn out to be. For example, John S. Dexter, investigating mutant notched wings of fruit flies (Dexter, 1914), could not have predicted the prominent role of the Notch cell surface receptor in cancer (Nowell and Radtke, 2017; Ranganathan et al., 2011). We do not have such foresight either. Nonetheless, it is worth pondering what more we can learn from model organisms, particularly in the era of human stem cells, organoids and facile genome editing using CRISPR/Cas9-based technologies.

## The historical impact of model organism research

Biological research was historically driven by curiosity about the natural world. The four model organisms discussed below – yeast, fruit fly, worm and sea urchin – came into use for practical reasons, such as the ease of rearing or their natural abundance. We describe examples of their contributions and discuss their attributes that have enabled investigators to ask questions that would be impractical or impossible to address using mammalian experimental systems.

### Yeast

We owe a great deal of our understanding of eukaryotic biology to two species of yeast: *Saccharomyces cerevisiae* (budding yeast) and *Schizosaccharomyces pombe* (fission yeast). They have been standout model organisms largely because of their simple life cycles and suitability for large-scale genetic analyses as described below. The relevance of such simple cells to the biology of complex organisms might not be obvious, but, as we discover time and time again, fundamental biological mechanisms are largely the same in simple and more complex organisms, as nicely illustrated by two influential studies (Table 1).

**Table 1.**
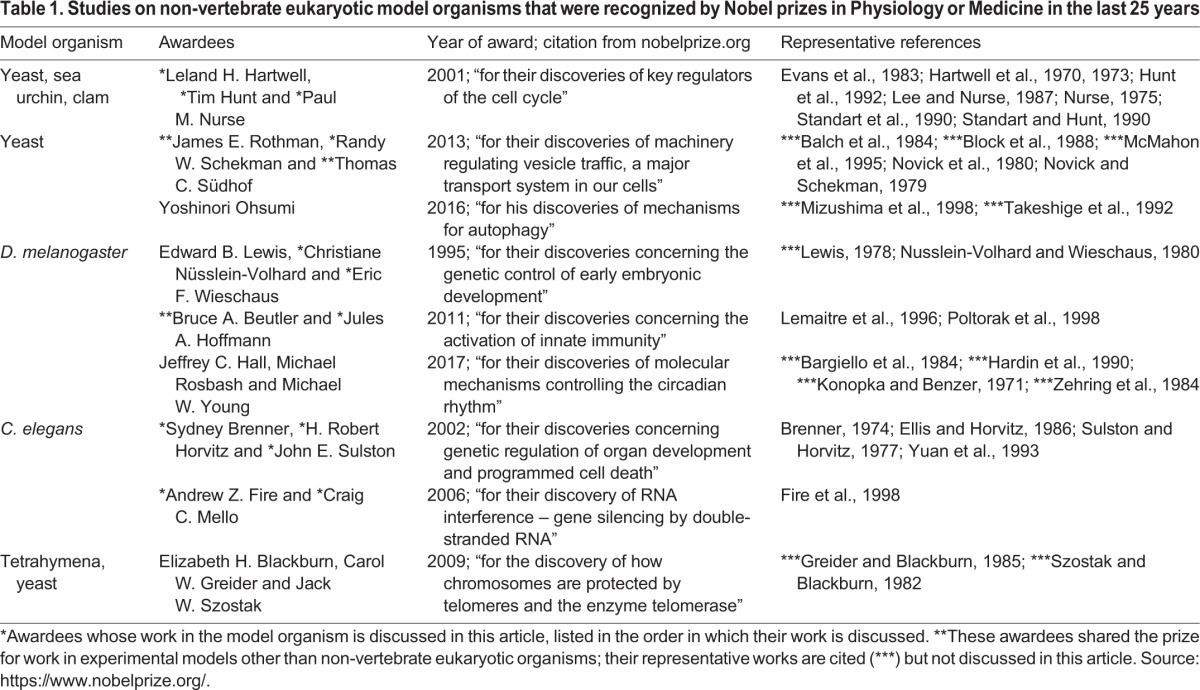
**Studies on non-vertebrate eukaryotic model organisms that were recognized by Nobel prizes in Physiology or Medicine in the last 25 years**

**Fig. 1.**
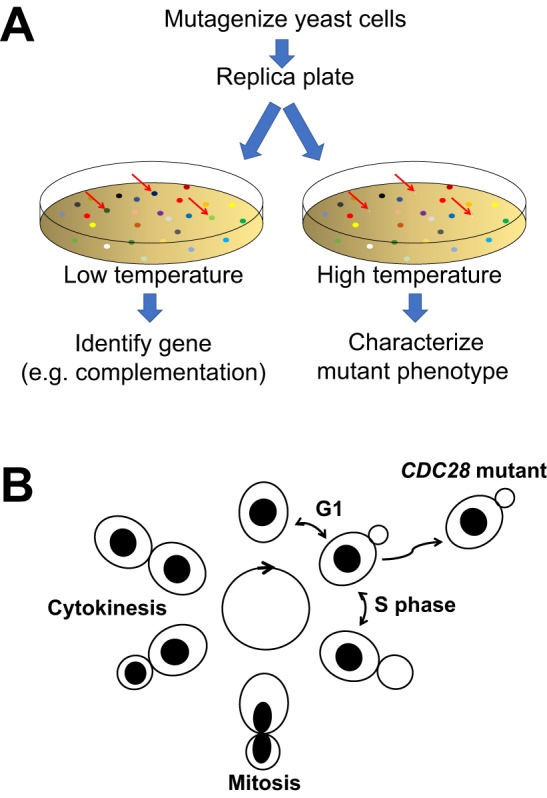
**The basis for a genetic screen for budding-yeast cell-cycle mutants.** (A) A genetic screen in yeast. Mutagenized yeast cells are cultured on replica plates. One plate is incubated at the low temperature to allow growth, whereas the other is incubated at a high temperature. Conditional mutants that fail to grow at the high temperature (arrows) are thus selected against when the plates are incubated at the high temperature. Microscopic analysis of the high-temperature plate identifies conditional mutants that are blocked in the cell cycle. These mutants, which retain the ability to grow at low temperature, are then isolated from the low-temperature plate for subsequent analysis, which includes complementation tests with wild-type genes to identify the gene responsible for the phenotype (Forsburg, 2001). (B) A budding-yeast cell cycle, modified from Hartwell et al., 1970, 1973. A mother cell produces a daughter by growing a bud that enlarges and eventually separates from the mother. Different stages of the cell cycle can be scored by the shape and size of the cells. For instance, the onset of DNA replication corresponds to the emergence of a bud. Mutants in *CDC28*, encoding the major cyclin-dependent kinase, arrest with a ‘small bud’, unable to enter a new cell cycle.

The first study asked a simple but fundamental question: which genes control how one cell becomes two? Leland Hartwell and his colleagues recognized that the way budding yeast grew and divided would allow the identification of mutants that are defective in the cell division cycle by simply observing the cells (Hartwell et al., 1970, 1973). The researchers took advantage of a useful feature of yeast. Because yeast can grow at different temperatures, one can isolate conditional mutants (Box 1) in which a gene product is active at a low temperature but becomes inactive at a high temperature. These temperature-sensitive conditional mutants could be grown and propagated at the low temperature and their defects examined after a shift to the high temperature. Because yeast cells are small and easy to grow, Hartwell and his colleagues were able to isolate many temperature-sensitive mutants (Fig. 1A). By simply observing cells at the high temperature, these researchers identified mutants that were blocked at different stages of the cell cycle (Fig. 1B). This genetic screen, combined with genetic tools such as epistasis analysis (Box 1), allowed Hartwell and colleagues to identify virtually all of the genes involved in controlling the cell division cycle, or the ‘CDC genes’ (Hartwell et al., 1970, 1973). A parallel analysis led by Paul Nurse and colleagues using similar methods identified the CDC genes in fission yeast (Lee and Nurse, 1987; Nurse, 1975).

The identification of the CDC genes defined a new problem: how do they work in concert to guide cell proliferation, perhaps the most basic of life's processes? Combining molecular biology and genetics in yeast, and with contributions from other model organisms (e.g. sea urchins; see below), the roles of the different CDC gene products were discovered. These advances came with the realization that the genes that control the cell cycle are largely conserved from yeast to human (e.g. Hartwell et al., 1970, 1973; Nurse, 1975). An exceptionally good example of this evolutionary conservation is the gene encoding cyclin-dependent kinase 1 (CDK1); the human *CDK1* gene was first identified by its ability to substitute for the fission yeast version and allow yeast that were mutant for *CDC2* (*CDK1* homolog) to continue dividing (Lee and Nurse, 1987). The regulation of CDK1 by inputs from many of the CDC genes governs the progress of the cell cycle. Moreover, the impact of yeast studies extended in many directions, raising questions such as: how is cell proliferation regulated during development; how do disruptions in this regulation derail proliferation control in cancer; and how can drugs modulating the function of CDC genes be used to treat cancer (Table 1)?

In another example, a simple observation in budding yeast ignited research in the field of protein secretion, which is of central importance to cellular function (Novick et al., 1980; Novick and Schekman, 1979). The isolation of the first secretory (SEC) mutants, in the laboratory of Randy Schekman, was made possible because of the realization that protein secretion is required to build a cell wall outside the cell membrane (Novick and Schekman, 1979). Subsequently, secretion-defective yeast mutants were recognized to be denser than their wild-type counterparts. This trait was used to isolate mutants in additional secretory pathway components using gradient sedimentation (Novick et al., 1980). Again, the genes identified provided the foundation for the mechanistic dissection of protein secretion. The resulting understanding of how an intricate system of membranous vesicles traffics proteins from the inside to the outside of cells also led to our understanding of other processes, such as how nerves signal to one another through the release of neurotransmitters. Yeast studies thus provided an entrée into a new area of cell biology (Table 1).

These genetic screens in yeasts (Fig. 1A) have changed the face of modern science and stimulated subsequent genetic analyses that allowed researchers to place genes in functional pathways even when insight into the biochemical properties of the gene products was lacking (e.g. Garvik et al., 1995). The resulting deep understanding of cell cycle regulation and membrane biology underpins many branches of modern biology and has guided medical research and biotechnology. For example, our understanding of protein secretion from yeast allowed us to manufacture recombinant human insulin for therapeutic purposes (Nielsen, 2013) and small-molecule inhibitors of WEE1, a regulator of CDK1, are being assessed in clinical trials for cancer (Matheson et al., 2016).

### Drosophila

**Fig. 2.**
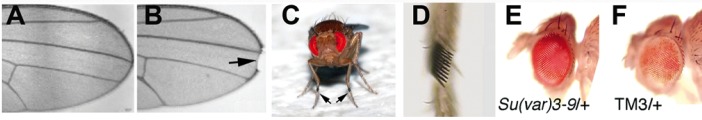
***Drosophila* mutants to illustrate landmark studies.** (A,B) Wild-type (A) and *Notch* mutant (B) wings showing notched (arrow) wing blades. Figure reproduced from Casso et al., 2011, with copyright permission from the publisher. (C,D) Sex combs on the front legs of a male *D. melanogaster* (arrows in C) are magnified in D. In *polycomb* mutants, anterior/posterior patterning is disrupted, resulting in sex combs appearing also on the middle and hind legs. Reproduced under a Creative Commons license from Wikicommons and with permission from http://flymove.uni-muenster.de. See also Weigmann et al., 2003. (E,F) Suppression of eye color variegation, from Qi et al., 2006. Variegation of eye color (F; juxtaposition of patches of white and red) is suppressed in heterozygotes of a mutation in *Su(var)3-9*, a gene that encodes an enzyme that methylates lysine 9 of histone H3 (E; uniformly red). TM3 is a balancer chromosome and serves as a ‘wild type’ control. Reproduced with copyright permission from the publisher.

How does one choose examples to adequately illustrate the impact of a model organism that is responsible for the confirmation of Mendelism (Box 1), the discovery of the first mutation and the demonstration that genetic traits are carried on chromosomes? The fruit fly *Drosophila melanogaster* has taught us many fundamental biological mechanisms, thanks to powerful genetic tools, which include phenotypic tractability, special ‘balancer’ chromosomes that allow long-term maintenance of lethal mutations, high fecundity and short life cycle, and the dedication of early fly geneticists, who learned to recognize every bristle on the fly or to detect the subtlest of deviations in wing shape or eye color. Many regulatory pathways studied in modern biology were either discovered in *Drosophila* or organized into regulatory circuits as a result of studies in *Drosophila*. For instance, although studies in mammals identified the Ras oncogenes (and others), we owe our understanding of their function to genetic studies of eye development in *Drosophila*, which revealed that Ras transmits signals from receptor tyrosine kinases (Simon et al., 1991; similar insights from worms are discussed below). Indeed, many signaling pathways of central interest to both normal biology and disease research bear the names of the genes discovered in *Drosophila* by scientists such as Christiane Nüsslein-Volhard, Eric F. Wieschaus and colleagues, including *hedgehog* (Nüsslein-Volhard and Wieschaus, 1980), *Notch* (Dexter, 1914; Fig. 2A,B) and *Toll* (Table 1). Toll was identified as a gene that functions in the establishment of dorsal/ventral polarity in the *Drosophila* embryo (Anderson et al., 1985a,b). The elegant genetic dissection of Toll function helped outline an elaborate pathway, which extends from extracellular signals to the activation of key conserved transcription factors, including NF-κB (called Dorsal in flies) and its relatives (Roth et al., 1989; Sen and Baltimore, 1986; Steward, 1989). Subsequently, it was shown that the same signaling system triggers an innate immune response in *Drosophila* through the work of Jules A. Hoffmann and his colleagues, and that this signaling system is the core component of innate immunity in mammals (Lemaitre et al., 1996; Poltorak et al., 1998). This pivotal discovery promoted a dramatic shift in the study of immunology from an emphasis on adaptive immunity to the more conserved innate system; the ratio of PubMed search results for ‘innate immunity’ vs ‘adaptive immunity’ was 1285:2860 in 1980 and 4423:2658 in 2017. Moreover, we now appreciate that NF-κB family transcription factors regulate almost all aspects of cell biology, from proliferation to inflammation and cell death (Zhang et al., 2017).

Unusual as it may seem, bristles on the leg of a fly helped us understand how the activity of genes is epigenetically regulated (Box 1). The regions that looked different within the chromosomes of moss and *Drosophila* were recognized in the early 1900s: densely stained heterochromatin and less densely stained euchromatin (Passarge, 1979). Continuing investigations showed that genes in heterochromatin were transcriptionally repressed and that this repression could be passed on to the next generation of cells (Brown and Nur, 1964). Surprisingly, the discovery of the underlying mechanisms of epigenetic inheritance involved studies of flies' bristles. The accurate formation of a set of leg bristles on male *Drosophila*, called the ‘sex comb’ for their role in mating (Fig. 2C,D), depends on the mechanisms that form heterochromatin. Mutations that result in the formation of additional sex combs identified a number of genes, with names like *Polycomb* and *extra sex combs* (Hannah-Alava, 1958; Reute and Spierer, 1992). Subsequent analyses demonstrated that these genes encode proteins that work together to regulate the addition or removal of chemical modifications to and from histones, the proteins that bind to and determine how DNA is packaged (Piunti and Shilatifard, 2016). Another assay in *Drosophila* allowed the isolation of additional chromatin-modulating genes. The red color of the fly eye requires the expression of the gene *white*, which encodes a pigment transporter (O'Hare et al., 1984; Sullivan et al., 1974). Translocation of the *white* gene near heterochromatin (for example by a chromosome rearrangement) can produce flies with eyes that show ‘variegation’, whereby white patches appear next to normally pigmented red patches (Fig. 2E,F; e.g. Qi et al., 2006). This process is known as position effect variegation (Box 1), and it happens because of changes in chromatin state, so that the *white* gene locus is in the heterochromatin state and thus transcriptionally inactive in some cells but not others, whereas the DNA sequence of the gene remains unchanged (Ebert et al., 2004). This phenotype illustrates how epigenetic regulation determines gene expression profiles, not on the basis of DNA sequence changes, but on the basis of chromatin state. Founder cells with an epigenetically inactive *white* gene produce daughter populations that form a white patch in the eye, whereas neighboring cells with a transcriptionally active *white* gene are red. Mutations that suppress the formation of these white patches are called suppressors of variegation, or Su(var) mutations, and their discovery identified additional chromatin-modulating genes and gene regulatory networks (Ebert et al., 2004; Reute and Spierer, 1992; Sinclair et al., 1983). For example, *Su(var)3-9* encodes a histone methyltransferase and *Su(var)2-5* encodes a protein, now called heterochromatin protein 1 (HP1), that binds to histones methylated by Su(var)3-9 (Aagaard et al., 1999; Ebert et al., 2004; Eissenberg and Elgin, 2014; Eissenberg et al., 1990; James and Elgin, 1986; Rea et al., 2000; Tschiersch et al., 1994). *Polycomb*, *Su(var)3-9* and *HP1* are just three of many genes with crucial roles in maintaining cell identity through epigenetic regulation of gene expression that were discovered in *Drosophila*. Their initial identification in *Drosophila* subsequently led to the characterization of their highly conserved counterparts in humans, where they also regulate transcription and influence many aspects of cell identity, physiology and pathogenesis (Allis and Jenuwein, 2016; Lanzuolo and Orlando, 2012; Piunti and Shilatifard, 2016; Schuettengruber et al., 2007; Timms et al., 2016).

### Caenorhabditis elegans

**Fig. 3.**
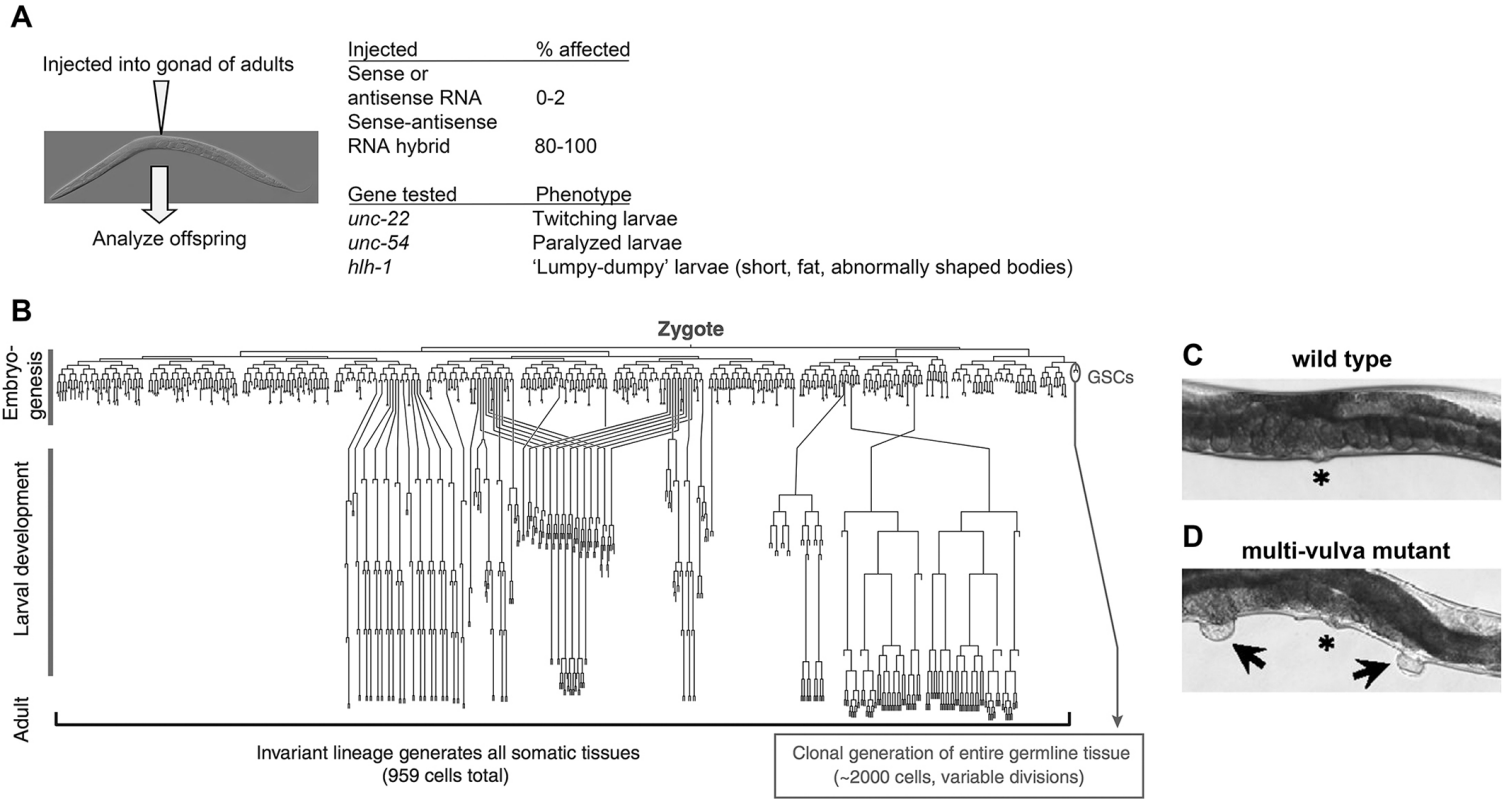
***C. elegans* cell-lineage map and multi-vulva mutants.** (A) Experiment and data that led to the discovery of RNAi, summarized from Fire et al., 1998. (B) Cell-lineage map of *C. elegans*. Reproduced under a Creative Commons license from Kimble and Seidel, 2013. (C,D) Wild-type and multi-vulva (Muv) mutant worms. * indicates the vulva; arrows point to ectopic vulvae. Figures modified and reproduced under a Creative Commons license from de la Cova and Greenwald, 2012. Specification of the vulval fate occurs through Ras/MAPK signaling (Kornfeld, 1997; Sundaram and Han, 1996). Mutants in which this pathway is misregulated can show the Muv phenotype (e.g. Gu et al., 1998), which led to the discovery of regulators of Ras/MAPK signaling.

Studies of *C. elegans* development, like those in *Drosophila*, led to breakthroughs in our understanding of cellular signaling. Early researchers of *C. elegans* took advantage of the transparent bodies of these small nematodes and used simple polarization or differential interference contrast (DIC) optics (Box 1) to track the fate of cells as an embryo developed (Brenner, 1974; Sulston and Horvitz, 1977). What they learned was the remarkable conservation of cell fate decisions from worm to worm. In fact, cell behavior is so rigid in this organism that Sydney Brenner, John Soulston, Bob Horvitz and colleagues were able to construct an accurate lineage map of all post-embryonic cell divisions and cell deaths (Fig. 3; Sulston and Horvitz, 1977). This was a gold mine for geneticists looking for mutants that deviated from the wild-type pattern of cell division and cell death. *C. elegan*s are hermaphrodites and can be mated to themselves (much like Mendel's garden peas) to produce progeny, typically in large numbers and in the expected Mendelian ratios (Box 1) (Brenner, 1974). These and the other benefits of *C. elegans* have led to many fundamental insights into biology, including the identification of the regulators of Ras signaling that arose from the first cloning of the *Ras* gene (Beitel et al., 1990; Han and Sternberg, 1990; Sternberg and Han, 1998), the discovery of RNA interference (RNAi; Fire et al., 1998; Table 1) and of the first microRNA (Lee et al., 1993; Wightman et al., 1993). The discovery of RNAi by Craig Mello, Andrew Fire and colleagues came on the heels of prior recognition that experimental addition of exogenous RNA could interfere with the expression of the corresponding endogenous genes in plants and worms (Sen and Blau, 2006). Some of the results, such as the finding that both sense and anti-sense strands had the same repressive effect, however, could not be explained by the prevailing model that exogenous RNA interfered with gene function simply by hybridization with the endogenous RNA. Instead, *C. elegans* studies showed that double-stranded RNA acted catalytically to suppress gene expression, thereby spearheading a field that now has wide applications in basic research, biomedicine and pest control, among others (Perrimon et al., 2010).

A key disease-relevant field that *C. elegans* genetics research pioneered is programmed cell death. Apoptosis, a form of programmed cell death, was first recognized in mammalian cells (Kerr et al., 1972) and it was known that enzymes called caspases were important for executing this cellular process. But it was not until Bob Horvitz and colleagues took a genetic approach to analyzing programmed cell death during worm development that our understanding of apoptosis truly advanced. These researchers exploited the invariant sequence of cell division and cell death during *C. elegans* development to screen for mutants in which too many or too few cells died (e.g. Ellis and Horvitz, 1986). Further studies led to the isolation of the gene that encodes the key caspase, *ced-3* (for *cell death mutant number 3*) (Yuan et al., 1993). *C. elegans* studies thus identified the genes that match mammalian caspase genes. Using genetic analysis in *C. elegans*, the enzymes that trigger and execute apoptosis were placed in an ordered pathway and their activators and repressors were identified (Metzstein et al., 1998). These efforts laid the foundation for a deeper understanding of apoptosis in mammalian systems (Table 1).

### Sea urchins

Classical forward genetics is not the only tool in the biologist's toolkit. Research into the embryonic development of marine invertebrates, such as the sea urchin, provides an excellent example of how combining biochemistry and cell biology results in a powerful discovery strategy. The practical advantages of using sea urchins attracted many researchers: low cost and relative ease of maintenance (Sluder, 2016); availability of a large quantity of sperm and eggs to enable biochemical studies; rapid, nearly synchronous cell cycles (40-90 min depending upon the temperature) that allow one to study multiple divisions in a short time; and optical transparency of the eggs of some species, useful for imaging. Sea urchin zygotes are remarkably robust and tolerate, for example, mechanical fragmentation or microinjection (Box 1) (Heasman, 2002). The publicly available genome sequence of some species provides access to genes of interest (Cameron et al., 2015; Sea Urchin Genome Sequencing Consortium et al., 2006) and genome editing in zygotes with CRISPR/Cas9 has been reported (Lin and Su, 2016).

Sea urchin gametes and zygotes have been studied since the late 1800s. Theodore Boveri used polyspermy (Box 1) to force a fertilized egg to divide its contents unequally into daughter cells. He analyzed the resulting abnormal zygotes and, with crisp deductive logic, Boveri considered and then discarded each cellular component as the bearer of genetic information until he identified the structures that we now call chromosomes (Boveri, 1974). Boveri's chromosome theory of inheritance was beautifully complemented by Mendel's work as re-discovered by Sutton (Sutton, 1903) and was amply supported by Morgan's experimental data from *Drosophila* (Morgan, 1911). Since then, many fundamental insights into cell biology have been gained using sea urchin gametes and zygotes. These include the identification of bindin, a protein that facilitates egg-sperm interactions for fertilization (Glabe and Vacquier, 1977; Vacquier and Moy, 1977); an understanding of how complex changes to the cytoskeleton and cell membranes are orchestrated to form a cleavage furrow (Box 1) (Pollard, 2004); the identification of gene regulatory networks that control early zygotic development (Davidson et al., 2002); understanding how centrosomes (Box 1) are assembled and duplicated (reviewed in Sluder, 2014); and the discovery of the cyclin proteins that drive the cell cycle in sea urchin zygotes and also in clam embryo extracts (Evans et al., 1983; Hunt et al., 1992; Standart et al., 1990; Standart and Hunt, 1990). We, the authors, still remember the excitement at the recognition that the cyclins that drive cleavage in sea urchin zygotes are homologous to the CDC proteins that drive the cell cycle in yeast (Booher and Beach, 1988; Hagan et al., 1988). These and many other findings from sea urchin models paved the way for subsequent discoveries in mammalian systems.

## Concluding remarks

As useful as model organisms are, they are not human, and have their limitations. Three-dimensional (3D) cell-culture model systems, such as organoids derived from human stem cells, have been much-heralded because they are the only human-cell-based model system to recapitulate the cellular complexity of tissues (Huch et al., 2017). Challenges exist, such as high cost and the phenotypic variability in traits, including organoid size, shape, cellular composition and 3D architecture, even when the organoids are produced under identical conditions (Huch et al., 2017), but these may be overcome as the technology matures. Lab-grown organoids should be useful for toxicity testing and for the generation of tissues for biobanks, but experts doubt that they will replace animal models as discovery tools (Bredenoord et al., 2017). We would go further to argue that organoids will not and should not replace non-vertebrate model organisms as discovery tools. We, the authors, speculate that the best use of organoids may be to transfer knowledge acquired in model organisms to humans. Given this position, what do we think are future discoveries that could be expected from curiosity-driven research in model organisms?

One obvious answer concerns the inner workings deep within the cell. For example, all cells, regardless of origin, need to make proteins, but also must get rid of proteins that are no longer needed. Genetic studies in budding yeast, complemented by biochemical and structural analyses, are providing us with exquisite details on how the protein machines responsible for destroying other proteins are assembled (e.g. Li et al., 2017; Sokolova et al., 2015). Likewise, we anticipate that studies in yeast and other model organisms may provide a deeper understanding of how proteins are synthesized, how they are modified with different chemical moieties, and how they are transported across and out of the cell. For such dissection of fundamental cellular processes, stem cells or organoids are neither required nor cost effective. Additionally, as with examples described in this article, we expect fundamental insights gleaned from model organisms to lead to better understanding of biology in all organisms, including humans.

Moving beyond cell biology to whole-animal biology, two examples illustrate the kind of new knowledge that can be gained from studying the biology of model organisms. An interesting question that has intrigued biologists for many years is how organisms, and the organs within them, attain their characteristic size. The *Drosophila* Hippo kinase pathway acts as a growth rheostat that fine-tunes the balance of proliferation and cell death during development, thereby helping organs attain their correct size and shape. It does so by regulating the transcription factor Yorkie (Yki; YAP in human), the targets of which include genes that regulate cell proliferation and apoptosis (Hariharan, 2015; Irvine and Harvey, 2015). The latest twist in the Hippo signaling tale involves *Drosophila* insulin-like peptide 8 (Dilp8), a peptide hormone secreted by injured tissues (Colombani et al., 2012; Garelli et al., 2012). Secreted Dilp8 accumulates in the circulatory system of the fly, from where it finds its way to the brain and stimulates neurons that subsequently repress the production of the molting hormone, thereby postponing the next stage in fly development. This signaling throughout the animal allows injured organs more time to grow and catch up in size relative to their undamaged counterparts. Strikingly, *dilp8* mutant flies lose coordinated organ growth, such that wings are mismatched in size (Garelli et al., 2012). *dilp8* was recently found to be a transcriptional target of the Hippo pathway (Boone et al., 2016). The current thinking is that Hippo not only fine-tunes growth within a tissue, but also acts via Dilp8 to delay development until organs at distant sites in a body are matched in size. When the pathway is functional, wing growth on opposite sides of the body is coordinated. The Hippo pathway also has emerged as an important regulator of the growth needed to repair injured tissues in a variety of organisms, including vertebrates (Hong et al., 2016; Patel et al., 2017; Zhang and Del Re, 2017). Given these roles, it is not surprising that the Hippo pathway – first discovered in *Drosophila* (Harvey et al., 2003; Justice et al., 1995; Pantalacci et al., 2003; Udan et al., 2003; Wu et al., 2003) – is also increasingly implicated in growth control in human cells and in the development of many human cancers (Hong et al., 2016; Patel et al., 2017). This example highlights how a strategy of ‘discovery first’ can be of tremendous benefit to biomedicine.

*C. elegans* provides another example of a model in which new insights about biology remain to be made. *C. elegans* hermaphrodites can produce sperm or egg, but not both simultaneously. A recently published study found that fatty acids (FAs) in the gut can alter the germline of hermaphrodites to stimulate production of either oocytes or sperm (Tang and Han, 2017). FAs exert their effect by altering the level of myristic acid (a form of FA) in the germline, which in turn affects the level of myristoylation, a process in which myristic acid is covalently attached to proteins. One protein controlled by myristoylation turns out to be the MAP kinase MPK-1, which determines germ-cell fate (Tang and Han, 2017). This biological phenomenon is conserved in *Caenorhabditis remanei*, a worm species that lives as male or female and not as a hermaphrodite, where the knocking down of the myristic acid-processing enzyme ACS-4 can masculinize genetically female worms into producing sperm.

What do these examples tell us about what the future might hold? They illustrate the complex interconnections between the germline and the soma through nutrients and signaling in worms, and a complex system of growth control and tissue repair that involves multiple organs and many secreted hormones in flies. They exemplify how model organisms can be used to understand how multiple organ systems interact inside an intact body, and to identify molecules that mediate these interactions. Such a deep understanding of biology and its complexities, we argue, will provide a basis for future breakthroughs in medicine. It will be a long time, if ever, before we can model complex interactions in organoids or cultured cells. Until then, it is curiosity-driven research in model organisms that will continue to satiate our appetite for understanding the wonderful and mysterious natural world that we live in.

## References

[DMM031203C1] Aagaard L., Laible G., Selenko P., Schmid M., Dorn R., Schotta G., Kuhfittig S., Wolf A., Lebersorger A., Singh P. B. et al. (1999). Functional mammalian homologues of the Drosophila PEV-modifier Su(var)3-9 encode centromere-associated proteins which complex with the heterochromatin component M31. *EMBO J.* 18, 1923-1938. 10.1093/emboj/18.7.1923 10202156 PMC1171278

[DMM031203C3] Allis C. D. and Jenuwein T. (2016). The molecular hallmarks of epigenetic control. *Nat. Rev. Genet.* 17, 487-500. 10.1038/nrg.2016.59 27346641

[DMM031203C4] Anderson K. V., Bokla L. and Nüsslein-Volhard C. (1985a). Establishment of dorsal-ventral polarity in the Drosophila embryo: the induction of polarity by the Toll gene product. *Cell* 42, 791-798. 10.1016/0092-8674(85)90275-2 3931919

[DMM031203C5] Anderson K. V., Jürgens G. and Nüsslein-Volhard C. (1985b). Establishment of dorsal-ventral polarity in the Drosophila embryo: genetic studies on the role of the Toll gene product. *Cell* 42, 779-789. 10.1016/0092-8674(85)90274-0 3931918

[DMM031203C112] Balch W. E., Dunphy W. G., Braell W. A. and Rothman J. E. (1984). Reconstitution of the transport of protein between successive compartments of the Golgi measured by the coupled incorporation of N-acetylglucosamine. *Cell* 39, 405-416. 10.1016/0092-8674(84)90019-9 6498939

[DMM031203C113] Bargiello T. A., Jackson F. R. and Young M. W. (1984). Restoration of circadian behavioural rhythms by gene transfer in Drosophila. *Nature* 312, 752-764. 10.1038/312752a0 6440029

[DMM031203C6] Beitel G. J., Clark S. G. and Horvitz H. R. (1990). Caenorhabditis elegans ras gene let-60 acts as a switch in the pathway of vulval induction. *Nature* 348, 503-509. 10.1038/348503a0 2123303

[DMM031203C115] Block M. R., Glick B. S., Wilcox C. A., Wieland F. T. and Rothman J. E. (1988). Purification of an N-ethylmaleimide-sensitive protein catalyzing vesicular transport. *Proc. Natl. Acad. Sci. U S A* 85, 7852-7856. 10.1073/pnas.85.21.7852 3186695 PMC282295

[DMM031203C7] Booher R. and Beach D. (1988). Involvement of cdc13+ in mitotic control in Schizosaccharomyces pombe: possible interaction of the gene product with microtubules. *EMBO J.* 7, 2321-2327. 2847913 10.1002/j.1460-2075.1988.tb03075.x PMC457096

[DMM031203C8] Boone E., Colombani J., Andersen D. S. and Léopold P. (2016). The Hippo signalling pathway coordinates organ growth and limits developmental variability by controlling dilp8 expression. *Nat. Commun.* 7, 13505 10.1038/ncomms13505 27874005 PMC5121414

[DMM031203C9] Boveri T. (1974). On multipolar mitosis as a means of analysis of the cell nucleus. In *Foundations of Experimental Embryology* (ed. Willier B. H. and Oppenheimer J. M.), pp. 74-97. New York: Hafner Press.

[DMM031203C10] Bredenoord A. L., Clevers H. and Knoblich J. A. (2017). Human tissues in a dish: the research and ethical implications of organoid technology. *Science* 355, eaaf9414 10.1126/science.aaf9414 28104841

[DMM031203C11] Brenner S. (1974). The genetics of Caenorhabditis elegans. *Genetics* 77, 71-94. 4366476 10.1093/genetics/77.1.71 PMC1213120

[DMM031203C12] Brown S. W. and Nur U. (1964). Heterochromatic chromosomes in the coccids. *Science* 145, 130-136. 10.1126/science.145.3628.130 14171547

[DMM031203C14] Cameron R. A., Kudtarkar P., Gordon S. M., Worley K. C. and Gibbs R. A. (2015). Do echinoderm genomes measure up? *Mar. Genomics* 22, 1-9. 10.1016/j.margen.2015.02.004 25701080 PMC4489978

[DMM031203C15] Casso D. J., Biehs B. and Kornberg T. B. (2011). A novel interaction between hedgehog and Notch promotes proliferation at the anterior-posterior organizer of the Drosophila wing. *Genetics* 187, 485-499. 10.1534/genetics.110.125138 21098717 PMC3030491

[DMM031203C16] Colombani J., Andersen D. S. and Leopold P. (2012). Secreted peptide Dilp8 coordinates Drosophila tissue growth with developmental timing. *Science* 336, 582-585. 10.1126/science.1216689 22556251

[DMM031203C17] Davidson E. H., Rast J. P., Oliveri P., Ransick A., Calestani C., Yuh C. H., Minokawa T., Amore G., Hinman V., Arenas-Mena C. et al. (2002). A genomic regulatory network for development. *Science* 295, 1669-1678. 10.1126/science.1069883 11872831

[DMM031203C18] de la Cova C. and Greenwald I. (2012). SEL-10/Fbw7-dependent negative feedback regulation of LIN-45/Braf signaling in C. elegans via a conserved phosphodegron. *Genes Dev.* 26, 2524-2535. 10.1101/gad.203703.112 23154983 PMC3505822

[DMM031203C19] Dexter J. S. (1914). The analysis of a case of continuous variation in Drosophila by a study of its linkage relations. *Am. Nat.* 48, 712-758. 10.1086/279446

[DMM031203C20] Dubnau J. and Tully T. (1998). Gene discovery in Drosophila: new insights for learning and memory. *Annu. Rev. Neurosci.* 21, 407-444. 10.1146/annurev.neuro.21.1.407 9530502

[DMM031203C21] Ebert A., Schotta G., Lein S., Kubicek S., Krauss V., Jenuwein T. and Reuter G. (2004). Su(var) genes regulate the balance between euchromatin and heterochromatin in Drosophila. *Genes Dev.* 18, 2973-2983. 10.1101/gad.323004 15574598 PMC534657

[DMM031203C22] Eissenberg J. C. and Elgin S. C. R. (2014). HP1a: a structural chromosomal protein regulating transcription. *Trends Genet.* 30, 103-110. 10.1016/j.tig.2014.01.002 24555990 PMC3991861

[DMM031203C23] Eissenberg J. C., James T. C., Foster-Hartnett D. M., Hartnett T., Ngan V. and Elgin S. C. (1990). Mutation in a heterochromatin-specific chromosomal protein is associated with suppression of position-effect variegation in Drosophila melanogaster. *Proc. Natl. Acad. Sci. USA* 87, 9923-9927. 10.1073/pnas.87.24.9923 2124708 PMC55286

[DMM031203C24] Ellis H. M. and Horvitz H. R. (1986). Genetic control of programmed cell death in the nematode C. elegans. *Cell* 44, 817-829. 10.1016/0092-8674(86)90004-8 3955651

[DMM031203C25] Evans T., Rosenthal E. T., Youngblom J., Distel D. and Hunt T. (1983). Cyclin: a protein specified by maternal mRNA in sea urchin eggs that is destroyed at each cleavage division. *Cell* 33, 389-396. 10.1016/0092-8674(83)90420-8 6134587

[DMM031203C26] Fang F. C. and Casadevall A. (2010). Lost in translation--basic science in the era of translational research. *Infect. Immun.* 78, 563-566. 10.1128/IAI.01318-09 20038540 PMC2812192

[DMM031203C27] Fatehullah A., Tan S. H. and Barker N. (2016). Organoids as an in vitro model of human development and disease. *Nat. Cell Biol.* 18, 246-254. 10.1038/ncb3312 26911908

[DMM031203C28] Fire A., Xu S. Q., Montgomery M. K., Kostas S. A., Driver S. E. and Mello C. C. (1998). Potent and specific genetic interference by double-stranded RNA in Caenorhabditis elegans. *Nature* 391, 806-811. 10.1038/35888 9486653

[DMM031203C29] Forsburg S. L. (2001). The art and design of genetic screens: yeast. *Nat. Rev. Genet.* 2, 659-668. 10.1038/35088500 11533715

[DMM031203C30] Garelli A., Gontijo A. M., Miguela V., Caparros E. and Dominguez M. (2012). Imaginal discs secrete insulin-like peptide 8 to mediate plasticity of growth and maturation. *Science* 336, 579-582. 10.1126/science.1216735 22556250

[DMM031203C31] Garvik B., Carson M. and Hartwell L. (1995). Single-stranded DNA arising at telomeres in cdc13 mutants may constitute a specific signal for the RAD9 checkpoint. *Mol. Cell. Biol.* 15, 6128-6138. 10.1128/MCB.15.11.6128 7565765 PMC230864

[DMM031203C32] Glabe C. G. and Vacquier V. D. (1977). Species specific agglutination of eggs by bindin isolated from sea urchin sperm. *Nature* 267, 836-838. 10.1038/267836a0 561310

[DMM031203C111] Greider C. W. and Blackburn E. H. (1985). Identification of a specific telomere terminal transferase activity in Tetrahymena extracts. *Cell* 43, 405-413. 10.1016/0092-8674(85)90170-9 3907856

[DMM031203C33] Gu T., Orita S. and Han M. (1998). Caenorhabditis elegans SUR-5, a novel but conserved protein, negatively regulates LET-60 Ras activity during vulval induction. *Mol. Cell. Biol.* 18, 4556-4564. 10.1128/MCB.18.8.4556 9671465 PMC109041

[DMM031203C34] Hagan I., Hayles J. and Nurse P. (1988). Cloning and sequencing of the cyclin-related cdc13+ gene and a cytological study of its role in fission yeast mitosis. *J. Cell Sci.* 91, 587-595. 2908246 10.1242/jcs.91.4.587

[DMM031203C35] Han M. and Sternberg P. W. (1990). let-60, a gene that specifies cell fates during C. elegans vulval induction, encodes a ras protein. *Cell* 63, 921-931. 10.1016/0092-8674(90)90495-Z 2257629

[DMM031203C36] Hand E., Mole B., Morello L., Tollefson J., Wadman M. and Witze A. (2013). A back seat for basic science. *Nature* 496, 277-279. 10.1038/496277a 23598316

[DMM031203C37] Hannah-Alava A. (1958). Developmental genetics of the posterior legs in Drosophila Melanogaster. *Genetics* 43, 878-905. 17247802 10.1093/genetics/43.5.878 PMC1209926

[DMM031203C114] Hardin P. E., Hall J. C. and Rosbash M. (1990). Feedback of the Drosophila period gene product on circadian cycling of its messenger RNA levels. *Nature* 343, 536-540. 10.1038/343536a0 2105471

[DMM031203C38] Hariharan I. K. (2015). Organ size control: lessons from Drosophila. *Dev. Cell* 34, 255-265. 10.1016/j.devcel.2015.07.012 26267393 PMC4547687

[DMM031203C39] Hartwell L. H., Culotti J. and Reid B. (1970). Genetic control of the cell-division cycle in yeast. I. Detection of mutants. *Proc. Natl. Acad. Sci. USA* 66, 352-359. 10.1073/pnas.66.2.352 5271168 PMC283051

[DMM031203C40] Hartwell L. H., Mortimer R. K., Culotti J. and Culotti M. (1973). Genetic control of the cell division cycle in yeast: V. Genetic analysis of cdc mutants. *Genetics* 74, 267-286. 17248617 10.1093/genetics/74.2.267 PMC1212945

[DMM031203C116] Harvey K. F., Pfleger C. M. and Hariharan I. K. (2003). The Drosophila Mst ortholog, hippo, restricts growth and cell proliferation and promotes apoptosis. *Cell* 114, 457-467. 10.1016/S0092-8674(03)00557-9 12941274

[DMM031203C41] Heasman J. (2002). Morpholino oligos: making sense of antisense? *Dev. Biol.* 243, 209-214. 10.1006/dbio.2001.0565 11884031

[DMM031203C42] Hong A. W., Meng Z. and Guan K.-L. (2016). The Hippo pathway in intestinal regeneration and disease. *Nat. Rev. Gastroenterol. Hepatol.* 13, 324-337. 10.1038/nrgastro.2016.59 27147489 PMC5642988

[DMM031203C43] Huch M., Knoblich J. A., Lutolf M. P. and Martinez-Arias A. (2017). The hope and the hype of organoid research. *Development* 144, 938-941. 10.1242/dev.150201 28292837

[DMM031203C44] Hunt T., Luca F. C. and Ruderman J. V. (1992). The requirements for protein synthesis and degradation, and the control of destruction of cyclins A and B in the meiotic and mitotic cell cycles of the clam embryo. *J. Cell Biol.* 116, 707-724. 10.1083/jcb.116.3.707 1530948 PMC2289306

[DMM031203C45] Irvine K. D. and Harvey K. F. (2015). Control of organ growth by patterning and hippo signaling in Drosophila. *Cold Spring Harb. Perspect. Biol.* 7, a019224 10.1101/cshperspect.a019224 PMC4448604 26032720

[DMM031203C46] James T. C. and Elgin S. C. (1986). Identification of a nonhistone chromosomal protein associated with heterochromatin in Drosophila melanogaster and its gene. *Mol. Cell. Biol.* 6, 3862-3872. 10.1128/MCB.6.11.3862 3099166 PMC367149

[DMM031203C117] Justice R. W., Zilian O., Woods D. F., Noll M. and Bryant P. J. (1995). The Drosophila tumor suppressor gene warts encodes a homolog of human myotonic dystrophy kinase and is required for the control of cell shape and proliferation. *Genes Dev.* 9, 534-546. 10.1101/gad.9.5.534 7698644

[DMM031203C47] Kenyon C. (2011). The first long-lived mutants: discovery of the insulin/IGF-1 pathway for ageing. *Philos. Trans. R. Soc. Lond. B Biol. Sci.* 366, 9-16. 10.1098/rstb.2010.0276 21115525 PMC3001308

[DMM031203C48] Kerr J. F. R., Wyllie A. H. and Currie A. R. (1972). Apoptosis: a basic biological phenomenon with wide-ranging implications in tissue kinetics. *Br. J. Cancer* 26, 239-257. 10.1038/bjc.1972.33 4561027 PMC2008650

[DMM031203C49] Kimble J. and Seidel H. C. (2013). C. elegans germline stem cells and their niche. In *StemBook [Internet]* (ed. L. Girard). Cambridge, MA: Harvard Stem Cell Institute. 24354021

[DMM031203C118] Konopka R. J. and Benzer S. (1971). Clock mutants of Drosophila melanogaster. *Proc. Natl. Acad. Sci. U S A* 68, 2112-2116. 10.1073/pnas.68.9.2112 5002428 PMC389363

[DMM031203C50] Kornfeld K. (1997). Vulval development in Caenorhabditis elegans. *Trends Genet.* 13, 55-61. 10.1016/S0168-9525(97)01005-6 9055606

[DMM031203C51] Kravitz E. A. and Fernandez M. P. (2015). Aggression in Drosophila. *Behav. Neurosci.* 129, 549-563. 10.1037/bne0000089 26348714

[DMM031203C52] Lanzuolo C. and Orlando V. (2012). Memories from the polycomb group proteins. *Annu. Rev. Genet.* 46, 561-589. 10.1146/annurev-genet-110711-155603 22994356

[DMM031203C53] Lee M. G. and Nurse P. (1987). Complementation used to clone a human homologue of the fission yeast cell cycle control gene cdc2. *Nature* 327, 31-35. 10.1038/327031a0 3553962

[DMM031203C54] Lee R. C., Feinbaum R. L. and Ambros V. (1993). The C. elegans heterochronic gene lin-4 encodes small RNAs with antisense complementarity to lin-14. *Cell* 75, 843-854. 10.1016/0092-8674(93)90529-Y 8252621

[DMM031203C119] Lee M. S., Kim J. I. and Ernst E. (2011). Is cupping an effective treatment? An overview of systematic reviews. *J. Acupunct. Meridian Stud.* 4, 1-4. 10.1016/S2005-2901(11)60001-0 21440874

[DMM031203C55] Lemaitre B., Nicolas E., Michaut L., Reichhart J.-M. and Hoffmann J. A. (1996). The dorsoventral regulatory gene cassette spätzle/Toll/cactus controls the potent antifungal response in Drosophila adults. *Cell* 86, 973-983. 10.1016/S0092-8674(00)80172-5 8808632

[DMM031203C110] Lewis E. B. (1978). A gene complex controlling segmentation in Drosophila. Nature 276, 565-570. 10.1038/276565a0 103000

[DMM031203C56] Li F., Tian G., Langager D., Sokolova V., Finley D. and Park S. (2017). Nucleotide-dependent switch in proteasome assembly mediated by the Nas6 chaperone. *Proc. Natl. Acad. Sci. USA* 114, 1548-1553. 10.1073/pnas.1612922114 28137839 PMC5321026

[DMM031203C57] Lin C.-Y. and Su Y.-H. (2016). Genome editing in sea urchin embryos by using a CRISPR/Cas9 system. *Dev. Biol.* 409, 420-428. 10.1016/j.ydbio.2015.11.018 26632489

[DMM031203C58] Matheson C. J., Backos D. S. and Reigan P. (2016). Targeting WEE1 Kinase in Cancer. *Trends Pharmacol. Sci.* 37, 872-881. 10.1016/j.tips.2016.06.006 27427153

[DMM031203C1111] McMahon H. T., Missler M., Li C. and Sudhof T. C. (1995). Complexins: cytosolic proteins that regulate SNAP receptor function. *Cell* 83, 111-119. 10.1016/0092-8674(95)90239-2 7553862

[DMM031203C59] McNutt M. (2015). Breakthrough to genome editing. *Science* 350, 1445 10.1126/science.aae0479 26680163

[DMM031203C60] Metzstein M. M., Stanfield G. M. and Horvitz H. R. (1998). Genetics of programmed cell death in C. elegans: past, present and future. *Trends Genet.* 14, 410-416. 10.1016/S0168-9525(98)01573-X 9820030

[DMM031203C61] Minogue K. and Wolinsky H. (2010). Lost in translation. *EMBO Rep.* 11, 93-96. 10.1038/embor.2009.282 20118990 PMC2828754

[DMM031203C1112] Mizushima N., Noda T., Yoshimori T., Tanaka Y., Ishii T., George M. D., Klionsky D. J., Ohsumi M. and Ohsumi Y. (1998). A protein conjugation system essential for autophagy. *Nature* 395, 395-398. 10.1038/26506 9759731

[DMM031203C62] Morgan T. H. (1911). The origin of five mutations in eye color in Drosophila and their modes of inheritance. *Science* 33, 534-537. 10.1126/science.33.849.534-a 17817675

[DMM031203C63] Nielsen J. (2013). Production of biopharmaceutical proteins by yeast: advances through metabolic engineering. *Bioengineered* 4, 207-211. 10.4161/bioe.22856 23147168 PMC3728191

[DMM031203C64] Novick P. and Schekman R. (1979). Secretion and cell-surface growth are blocked in a temperature-sensitive mutant of Saccharomyces cerevisiae. *Proc. Natl. Acad. Sci. USA* 76, 1858-1862. 10.1073/pnas.76.4.1858 377286 PMC383491

[DMM031203C65] Novick P., Field C. and Schekman R. (1980). Identification of 23 complementation groups required for post-translational events in the yeast secretory pathway. *Cell* 21, 205-215. 10.1016/0092-8674(80)90128-2 6996832

[DMM031203C66] Nowell C. S. and Radtke F. (2017). Notch as a tumour suppressor. *Nat. Rev. Cancer* 17, 145-159. 10.1038/nrc.2016.145 28154375

[DMM031203C67] Nurse P. (1975). Genetic control of cell size at cell division in yeast. *Nature* 256, 547-551. 10.1038/256547a0 1165770

[DMM031203C68] Nüsslein-Volhard C. and Wieschaus E. (1980). Mutations affecting segment number and polarity in Drosophila. *Nature* 287, 795-801. 10.1038/287795a0 6776413

[DMM031203C69] O'Hare K., Murphy C., Levis R. and Rubin G. M. (1984). DNA sequence of the white locus of Drosophila melanogaster. *J. Mol. Biol.* 180, 437-455. 10.1016/0022-2836(84)90021-4 6084717

[DMM031203C1115] Pantalacci S., Tapon N. and Leopold P. (2003). The Salvador partner Hippo promotes apoptosis and cell-cycle exit in Drosophila. *Nat. Cell Biol.* 5, 921-917 10.1038/ncb1051 14502295

[DMM031203C70] Passarge E. (1979). Emil Heitz and the concept of heterochromatin: longitudinal chromosome differentiation was recognized fifty years ago. *Am. J. Hum. Genet.* 31, 106-115. 377956 PMC1685768

[DMM031203C71] Patel S. H., Camargo F. D. and Yimlamai D. (2017). Hippo signaling in the liver regulates organ size, cell fate, and carcinogenesis. *Gastroenterology* 152, 533-545. 10.1053/j.gastro.2016.10.047 28003097 PMC5285449

[DMM031203C72] Perrimon N., Ni J.-Q. and Perkins L. (2010). In vivo RNAi: today and tomorrow. *Cold Spring Harb. Perspect. Biol.* 2, a003640 10.1101/cshperspect.a003640 20534712 PMC2908776

[DMM031203C73] Piunti A. and Shilatifard A. (2016). Epigenetic balance of gene expression by Polycomb and COMPASS families. *Science* 352, aad9780 10.1126/science.aad9780 27257261

[DMM031203C74] Pollard T. D. (2004). Ray Rappaport chronology: twenty-five years of seminal papers on cytokinesis in the Journal of Experimental Zoology. *J. Exp. Zool. A Comp. Exp. Biol.* 301A, 9-14. 10.1002/jez.a.20000 14695684

[DMM031203C75] Poltorak A., He X., Smirnova I., Liu M. Y., Van Huffel C., Du X., Birdwell D., Alejos E., Silva M., Galanos C. et al. (1998). Defective LPS signaling in C3H/HeJ and C57BL/10ScCr mice: mutations in Tlr4 gene. *Science* 282, 2085-2088. 10.1126/science.282.5396.2085 9851930

[DMM031203C76] Porter J. R. (1961). Louis PASTEUR; achievements and disappointments, 1861. *Bacteriol. Rev.* 25, 389-403. 14037390 10.1128/br.25.4.389-403.1961 PMC441122

[DMM031203C77] Qi D., Jin H., Lilja T. and Mannervik M. (2006). Drosophila Reptin and other TIP60 complex components promote generation of silent chromatin. *Genetics* 174, 241-251. 10.1534/genetics.106.059980 16816423 PMC1569795

[DMM031203C78] Ranganathan P., Weaver K. L. and Capobianco A. J. (2011). Notch signalling in solid tumours: a little bit of everything but not all the time. *Nat. Rev. Cancer* 11, 338-351. 10.1038/nrc3035 21508972

[DMM031203C79] Rea S., Eisenhaber F., O'Carroll D., Strahl B. D., Sun Z.-W., Schmid M., Opravil S., Mechtler K., Ponting C. P., Allis C. D. et al. (2000). Regulation of chromatin structure by site-specific histone H3 methyltransferases. *Nature* 406, 593-599. 10.1038/35020506 10949293

[DMM031203C80] Reute G. and Spierer P. (1992). Position effect variegation and chromatin proteins. *BioEssays* 14, 605-612. 10.1002/bies.950140907 1365916

[DMM031203C81] Roth S., Stein D. and Nüsslein-Volhard C. (1989). A gradient of nuclear localization of the dorsal protein determines dorsoventral pattern in the Drosophila embryo. *Cell* 59, 1189-1202. 10.1016/0092-8674(89)90774-5 2688897

[DMM031203C82] Schuettengruber B., Chourrout D., Vervoort M., Leblanc B. and Cavalli G. (2007). Genome regulation by polycomb and trithorax proteins. *Cell* 128, 735-745. 10.1016/j.cell.2007.02.009 17320510

[DMM031203C83] Sea Urchin Genome Sequencing Consortium, Sodergren E. Weinstock G. M. Davidson E. H. Cameron R. A. Gibbs R. A. Angerer R. C. Angerer L. M. Arnone M. I. Burgess D. R. et al. (2006). The genome of the sea urchin Strongylocentrotus purpuratus. *Science* 314, 941-952. 10.1126/science.1133609 17095691 PMC3159423

[DMM031203C84] Sen R. and Baltimore D. (1986). Multiple nuclear factors interact with the immunoglobulin enhancer sequences. *Cell* 46, 705-716. 10.1016/0092-8674(86)90346-6 3091258

[DMM031203C85] Sen G. L. and Blau H. M. (2006). A brief history of RNAi: the silence of the genes. *FASEB J.* 20, 1293-1299. 10.1096/fj.06-6014rev 16816104

[DMM031203C86] Simon M. A., Bowtell D. D. L., Dodson G. S., Laverty T. R. and Rubin G. M. (1991). Ras1 and a putative guanine nucleotide exchange factor perform crucial steps in signaling by the sevenless protein tyrosine kinase. *Cell* 67, 701-716. 10.1016/0092-8674(91)90065-7 1934068

[DMM031203C87] Sinclair D. A. R., Mottus R. C. and Grigliatti T. A. (1983). Genes which suppress position-effect variegation in Drosophila melanogaster are clustered. *Mol. Gen. Genet.* 191, 326-333. 10.1007/BF00334834

[DMM031203C88] Sluder G. (2014). One to only two: a short history of the centrosome and its duplication. *Philos. Trans. R. Soc. Lond. B Biol. Sci.* 369 10.1098/rstb.2013.0455 PMC4113099 25047609

[DMM031203C89] Sluder G. (2016). Using sea urchin gametes and zygotes to investigate centrosome duplication. *Cilia* 5, 20 10.1186/s13630-016-0043-3 27602205 PMC5011938

[DMM031203C90] Sokolova V., Li F., Polovin G. and Park S. (2015). Proteasome activation is mediated via a functional switch of the Rpt6 C-terminal tail following chaperone-dependent assembly. *Sci. Rep.* 5, 14909 10.1038/srep14909 26449534 PMC4598862

[DMM031203C91] Standart N. and Hunt T. (1990). Control of translation of masked mRNAs in clam oocytes. *Enzyme* 44, 106-119. 10.1159/000468751 2151945

[DMM031203C92] Standart N., Dale M., Stewart E. and Hunt T. (1990). Maternal mRNA from clam oocytes can be specifically unmasked in vitro by antisense RNA complementary to the 3′-untranslated region. *Genes Dev.* 4, 2157-2168. 10.1101/gad.4.12a.2157 2148535

[DMM031203C93] Sternberg P. W. and Han M. (1998). Genetics of RAS signaling in C. elegans. *Trends Genet.* 14, 466-472. 10.1016/S0168-9525(98)01592-3 9825675

[DMM031203C94] Steward R. (1989). Relocalization of the dorsal protein from the cytoplasm to the nucleus correlates with its function. *Cell* 59, 1179-1188. 10.1016/0092-8674(89)90773-3 2598266

[DMM031203C95] Sullivan D. T., Grillo S. L. and Kitos R. J. (1974). Subcellular localization of the first three enzymes of the ommochrome synthetic pathway in Drosophila melanogaster. *J. Exp. Zool.* 188, 225-233. 10.1002/jez.1401880210 4133038

[DMM031203C96] Sulston J. E. and Horvitz H. R. (1977). Post-embryonic cell lineages of the nematode, Caenorhabditis elegans. *Dev. Biol.* 56, 110-156. 10.1016/0012-1606(77)90158-0 838129

[DMM031203C97] Sundaram M. and Han M. (1996). Control and integration of cell signaling pathways during C. elegans vulval development. *BioEssays* 18, 473-480. 10.1002/bies.950180609 8787535

[DMM031203C98] Sutton W. S. (1903). The chromosomes in heredity. *Biol. Bull.* 4, 231-250. 10.2307/1535741

[DMM031203C1113] Szostak J. W. and Blackburn E. H. (1982). Cloning yeast telomeres on linear plasmid vectors. *Cell* 29, 245-255. 10.1016/0092-8674(82)90109-X 6286143

[DMM031203C1114] Takeshige K., Baba M., Tsuboi S., Noda T. and Ohsumi Y. (1992). Autophagy in yeast demonstrated with proteinase-deficient mutants and conditions for its induction. *J. Cell Biol.* 119, 301-311. 10.1083/jcb.119.2.301 1400575 PMC2289660

[DMM031203C99] Tang H. and Han M. (2017). Fatty acids regulate germline sex determination through ACS-4-dependent myristoylation. *Cell* 169, 457-469.e13. 10.1016/j.cell.2017.03.049 28431246

[DMM031203C100] Timms R. T., Tchasovnikarova I. A. and Lehner P. J. (2016). Position-effect variegation revisited: HUSHing up heterochromatin in human cells. *BioEssays* 38, 333-343. 10.1002/bies.201500184 26853531

[DMM031203C101] Tschiersch B., Hofmann A., Krauss V., Dorn R., Korge G. and Reuter G. (1994). The protein encoded by the Drosophila position-effect variegation suppressor gene Su(var)3-9 combines domains of antagonistic regulators of homeotic gene complexes. *EMBO J.* 13, 3822-3831. 7915232 10.1002/j.1460-2075.1994.tb06693.x PMC395295

[DMM031203C1116] Udan R. S., Kango-Singh M., Nolo R., Tao C. and Halder G. (2003). Hippo promotes proliferation arrest and apoptosis in the Salvador/Warts pathway. *Nat. Cell Biol.* 5, 914-920. 10.1038/ncb1050 14502294

[DMM031203C102] Vacquier V. D. and Moy G. W. (1977). Isolation of bindin: the protein responsible for adhesion of sperm to sea urchin eggs. *Proc. Natl. Acad. Sci. USA* 74, 2456-2460. 10.1073/pnas.74.6.2456 267939 PMC432191

[DMM031203C103] Weigmann K., Klapper R., Strasser T., Rickert C., Technau G., Jäckle H., Janning W. and Klämbt C. (2003). FlyMove--a new way to look at development of Drosophila. *Trends Genet.* 19, 310-311. 10.1016/S0168-9525(03)00050-7 12801722

[DMM031203C104] Wightman B., Ha I. and Ruvkun G. (1993). Posttranscriptional regulation of the heterochronic gene lin-14 by lin-4 mediates temporal pattern formation in C. elegans. *Cell* 75, 855-862. 10.1016/0092-8674(93)90530-4 8252622

[DMM031203C1117] Wu S., Huang J., Dong J. and Pan D. (2003). hippo encodes a Ste-20 family protein kinase that restricts cell proliferation and promotes apoptosis in conjunction with salvador and warts. *Cell* 114, 445-456. 10.1016/S0092-8674(03)00549-X 12941273

[DMM031203C105] Yuan J., Shaham S., Ledoux S., Ellis H. M. and Horvitz H. R. (1993). The C. elegans cell death gene ced-3 encodes a protein similar to mammalian interleukin-1 beta-converting enzyme. *Cell* 75, 641-652. 10.1016/0092-8674(93)90485-9 8242740

[DMM031203C1118] Zehring W. A., Wheeler D. A., Reddy P., Konopka R. J., Kyriacou C. P., Rosbash M. and Hall J. C. (1984). P-element transformation with period locus DNA restores rhythmicity to mutant, arrhythmic Drosophila melanogaster. *Cell* 39, 369-376. 10.1016/0092-8674(84)90015-1 6094014

[DMM031203C106] Zhang Y. and Del Re D. P. (2017). A growing role for the Hippo signaling pathway in the heart. *J. Mol. Med. (Berl.)* 95, 465-472. 10.1007/s00109-017-1525-5 28280861 PMC5404975

[DMM031203C107] Zhang Q., Lenardo M. J. and Baltimore D. (2017). 30 years of NF-kappaB: a blossoming of relevance to human pathobiology. *Cell* 168, 37-57. 10.1016/j.cell.2016.12.012 28086098 PMC5268070

